# The association between specific IgE antibodies to component allergens and allergic symptoms on dog and cat exposure among Korean pet exhibition participants^☆^^☆☆^

**DOI:** 10.1016/j.waojou.2022.100709

**Published:** 2022-10-12

**Authors:** Sung-Yoon Kang, Min-Suk Yang, Magnus P. Borres, Mats Andersson, Sang Min Lee, Sang Pyo Lee

**Affiliations:** a Department of Internal Medicine, Gachon University Gil Medical Center, Incheon, Republic of Korea; b Department of Internal Medicine, SMG-SNU Boramae Medical Center, Seoul, Republic of Korea; c Department of Maternal and Child Health, Uppsala University, Uppsala, Sweden; d ImmunoDiagnostic Division, Thermo Fisher Scientific, Uppsala, Sweden

**Keywords:** Allergy, Component-resolved diagnostics, Cat, Dog, Furry pet animal, Can f, Canis familiaris (dog), CRD, Component resolved diagnostics, Fel d, Felis domesticus (cat), FeNO, Fractional dose of exhaled nitric oxide, IQR, Interquartile range, MWD, Mean wheal diameter, PD20, Provocative dose of methacholine causing a 20% drop in forced expiratory volume in 1 second, sIgE, Allergen-specific immunoglobulin E, sIgG4, Allergen-specific immunoglobulin G4, SPT, Skin prick test

## Abstract

**Background:**

Component resolved diagnostics (CRD) in dog and cat allergy is not sufficiently investigated, especially regarding new components such as Can f 4, Can f 6, and Fel d 7. The purpose of this study is to evaluate the potential role of CRD with new components in predicting allergic symptoms on dog and cat exposure.

**Methods:**

Among 552 Korean adults who participated in a pet exhibition and completed questionnaires regarding exposure to dog or cat and allergic symptoms, 522 were venipunctured for measurement of IgE and IgG4 antibody concentration against dog and cat dander extract and underwent skin prick test (SPT). In 238 individuals who were sensitized for both dog and cat dander extract, the dog IgE components (Can f 1–6) and the cat components (Fel d 1/2/4/7) were analyzed.

**Results:**

An increasing number of sensitizing components was associated with the likelihood of having any allergic symptoms (*P* < 0.001 for dog and *P* < 0.01 for cat), and those of asthma (*P* < 0.01 for dog and *P* < 0.05 for cat) and rhinoconjunctivitis (*P* < 0.001 for dog and *P* < 0.05 for cat). Pairwise correlation of IgE levels was *r* = 0.56 (*P* < 0.001) for Can f 6 and Fel d 4, *r* = 0.74 (*P* < 0.001) for Can f 1 and Fel d 7 and *r* = 0.84 (*P* < 0.001) for Can f 3 and Fel d 2.

**Conclusions:**

Polysensitization to dog and cat allergen components is associated with high likelihood of having allergic symptoms during exposure to dogs and cats. Cross-reactivity between dog and cat allergen components is also identified. CRD has a potential in fine-tuning prediction for allergic symptoms on dog and cat exposure.

## Introduction

Allergy to cats and dogs has been recognized for many years as a major risk factor for the development of asthma and allergic rhinitis.**^1^** The prevalence of allergy to dog and cat as well as sensitization to those animals seem to have increased during recent years.^2^, ^3^, ^4^

The diagnosis of cat allergy has proven rather uncomplicated, probably since most patients react to the main protein, Fel d 1.**^5^** The diagnosis of dog allergy is more challenging; self-reporting misclassifies allergic status in many patients.**^6^** There is a clinical need for improved extracts for pet allergies because both standardized and unstandardized extracts are used.**^7^** Wintersand et al found recently that there is a great variation of dog allergens in natural extracts raising questions of source, sampling, processing, and ultimately of standardization and minimum allergen levels for accurate diagnosis and treatment.**^8^** Protein content in skin prick test (SPT) extracts for dogs can vary up to 20-fold within different manufacturers.**^9^**

Component-resolved diagnostics (CRD) is beginning to gain greater recognition for pet allergy diagnostics.**^10^** CRD identifies specific immunoglobulin E (sIgE) responses to certain molecular targets and several molecular allergen components have been identified for both cat and dog. However, this dimension also increases the complexity of diagnostics as some components may represent cross-reactivity. Studies of the utility of sIgE testing for pet components in adults are limited. Suzuki et al et al found that sensitization to Can f 5 was the most frequent component in a random population (3%) and Can f 1 in an asthma sample (14%).**^11^** Tsolakis et al found that levels of IgE to lipocalin (Fel d 4) and serum albumin (Fel d 2), but not to secretoglobin (Fel d 1) or cat extract, were independently associated with type-2 biomarkers and total IgE in young asthmatics.**^12^** Hemmer et al recently found that individual sensitization patterns strongly mirrored current or previous pet ownership except for Fel d 1 which regularly caused sensitization also in non-owners.**^13^**

Nwaru et al found that pet allergen component sensitization, particularly polysensitization, was associated with increased fractional exhaled nitric oxide (FENO) and eosinophil levels, and associated with lower PD20 methacholine reactivity values.**^14^** This was interpreted that sensitization to furry animal allergen components is an important predictor of particularly eosinophilic inflammatory markers of asthma severity. Polysensitization to pet components increased the risk of having asthma almost double than in allergic rhinitis. Roberts et al applied clustering methods to explore the connectivity structure of component-sIgE, and differences in component-sIgE interactions between severe and mild/moderate asthma.**^15^** They found that participants with severe asthma had higher connectivity among components (ie, more connections between different components), but these connections were weaker. The mild/moderate network had fewer connections, but the connections were stronger. Interestingly, connections among animal components showed higher correlations in severe asthma in adults than in mild to moderate asthma.

There is a need to further explore the clinical relevance of sensitization to cat and dog allergen components in different populations. The aim of this study was to analyze the patterns of IgE reactivity to dog and cat allergen components among Korean adults participating in a pet exhibition who had suffered from allergic symptoms on dog and cat exposure.

## Methods

### Study subjects

We enrolled participants who attended a pet exhibition entitled the “Korea Pet (KOPET) Show” during September 2018. Details of the study has been presented elsewhere.**^16^** Subjects were asked to answer a questionnaire regarding exposure and symptoms to cat and dog after informed consents were obtained. Age, gender, and current allergic diseases were documented. The type of allergic symptoms that occurred immediately on cat or dog exposure was collected. The included symptoms were those of allergic conjunctivitis (red, swollen and itchy eyes with or without tears), allergic rhinitis (watery rhinorrhea, sneezing, nasal congestion, postnasal drip, and itchy sensation on nose, ears, or palatine), asthma (dyspnea accompanied by wheezing and chest discomfort that fluctuated), skin allergy (itchy sensation on skin, hives, angioedema and/or eruptions on their skin), and cough.^16^^,^^17^ The subject was classified as pet allergic if they experienced any of these symptoms during direct exposure to dog or cat.^16^^,^^17^

### Skin prick test

Participants underwent skin prick test (SPT) using dog and cat dander allergen extracts from 3 different companies (HollisterStier, Spokane, WA, USA; Lofarma, Milan, Italy; and Allergy Therapeutics, Worthing, West Sussex, UK). SPT was performed on the forearms by trained investigators using sharp-pointed lancets. Histamine was used as positive control (10 mg/mL, HollisterStier; 1%, Lofarma; 0.1%, Allergy Therapeutics). SPT using glycerinated saline was the negative control. The skin test results were interpreted after 15 min by measuring the mean wheal diameter (MWD) induced by each allergen. The SPT results were regarded as positive if the MWD induced by one or more of three commercially available dog or cat dander allergens was ≥3 mm.

### Serologic analysis

Samples of venous blood were collected (*n* = 522), and IgE and IgG4 antibody concentration against dog dander extract (e5) and cat dander extract (e1) were measured. If sensitization to both dog and cat dander allergens was proven by SPT or specific IgE measurement (*n* = 238), the dog components (Can f 1, Can f 2, Can f 3, Can f 4, Can f 5, and Can f 6) and the cat components (Fel d 1, Fel d 2, Fel d 4, and Fel d 7), were analyzed by using ImmunoCAP (Thermo Fisher Scientific, Uppsala, Sweden). All IgE determinations were analyzed by using the ImmunoCAP System (Phadia AB/Thermo Fisher Scientific), according to the manufacturer's instructions. Results are presented as kilounits of allergen per liter, where the cutoff for the presence of allergen specific IgE was 0.10 kU_A_/L or greater, which is the cutoff level in clinical settings.**^16^**

Circulating IgG4 ab specific to cat and dog was determined in plasma (ImmunoCAP® 1000; Phadia AB) and no cutoff definition exists for sIgG 4.

### Statistical analysis

Continuous data and ordered categorical data with more than 2 levels were compared between groups by exact Wilcoxon test and categorical data with two levels were compared by Fisher's exact test. Correlation coefficients were calculated using Spearman's method. Two-sided p-values less than 0.05 were considered statistically significant. All statistical analyses were performed using R version 4.0.1.

### Ethics statement

The study was conducted in accordance with good clinical practice (GCP) guidelines. The protocol of this study was reviewed and approved by the institutional review board of our institution, Gachon University Gil Medical Center.

## Results

**Fig. 1:**
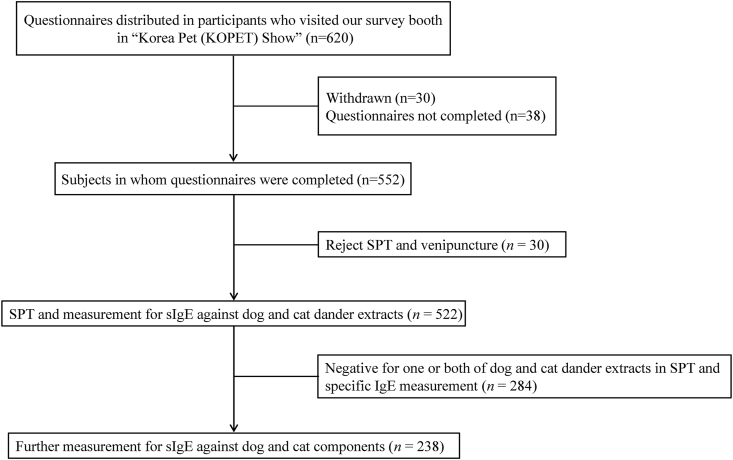
Flowchart of the study population. sIgE, specific immunoglobulin E

Six hundred and twenty subjects were willing to participate in the study (Fig. 1). However, 30 subjects withdrew their enroll and 38 returned an incomplete questionnaire. Thus, the study group consisted of 552 individuals that fulfilled the study criteria. Thirty subjects rejected SPT and venipuncture. Hence, data on antibody testing are available for 522 subjects. Two hundred and thirty-eight subjects were proven to be sensitized for both dog and cat dander allergens by SPT or specific IgE measurement and the following results are based on this subgroup.

### Baseline demographics

Demographic characteristics are shown in Table 1. The median age of the subjects was 28.0 years (interquartile range (IQR) 24.0–34.0 years), and females were in the majority (78.6%). Twenty-eight (11.8%) subjects had allergic symptoms upon both dog and cat contact, while 46 (19.3%) and 64 (26.9%) had symptoms only upon dog and cat contact, respectively. One hundred (42.0%) subjects did not suffer from allergic symptoms upon dog or cat exposure. For both dog and cat allergies, rhinitis was the most common symptom, followed by conjunctivitis, skin allergy, cough, and asthma. All subjects were proven to be sensitized to both dog and cat dander allergens by SPT or serum specific IgE measurement.

**Table 1:** Demographic characteristics of the study subjects (*n* = 238)

	All subjects (*n* = 238)	Allergy to both dog and cat (*n* = 28)	Allergy only to dog (*n* = 46)	Allergy only to cat (*n* = 64)	No allergy to dog or cat (*n* = 100)
Age (years)	28.0 [24.0, 34.0]	29.0 [26.3, 36.5]	28.5 [24.8, 34.3]	28.5 [24.8, 34.3]	28.5 [23.0, 35.8]
Female	187 (78.6)	26 (92.9)	31 (67.4)	51 (79.7)	79 (79.0)
Type of dog allergy					
Allergic rhinitis	60 (25.2)	21 (75.0)	39 (84.8)		
Allergic conjunctivitis	54 (22.7)	18 (64.3)	36 (78.3)		
Skin allergy	42 (17.6)	13 (46.4)	29 (63.0)		
Cough	25 (10.5)	8 (28.6)	17 (37.0)		
Asthma	15 (6.3)	6 (21.4)	9 (19.6)		
Type of cat allergy					
Allergic rhinitis	73 (30.7)	21 (75.0)		52 (81.3)	
Allergic conjunctivitis	71 (29.8)	10 (67.9)		52 (81.3)	
Skin allergy	47 (19.7)	14 (50.0)		33 (51.6)	
Cough	26 (10.9)	9 (32.1)		17 (26.6)	
Asthma	17 (7.1)	6 (21.4)		11 (17.2)	
Sensitization to dog proven by					
Skin prick test	114 (47.9)	11 (39.3)	**27 (58.7)**^a^	33 (51.6)	43 (43.0)
Specific IgE	198 (83.2)	**26 (92.9)**^a^	**45 (97.8)**^c^	52 (81.3)	75 (75.0)
Skin prick test or specific IgE	238 (100.0)	28 (100.0)	46 (100.0)	64 (100.0)	100 (100.0)
Sensitization to cat proven by					
Skin prick test	134 (56.3)	14 (50.0)	23 (50.0)	**42 (65.6)**^a^	55 (55.0)
Specific IgE	206 (86.6)	**28 (100.0)**^b^	37 (80.4)	**63 (98.4)**^c^	78 (78.0)
Skin prick test or specific IgE	238 (100.0)	28 (100.0)	46 (100.0)	64 (100.0)	100 (100.0)
Serum concentration, kU_A_/L					
Specific IgE to dog dander	0.80 [0.15, 3.43]	**2.49 [1.15, 9.36]**^c^	**3.09 [1.19, 23.3]**^c^	0.50 [0.13, 2.49]	0.26 [0.09, 1.31]
Specific IgE to cat dander	1.36 [0.25, 6.49]	**5.00 [0.94, 12.95]**^c^	0.53 [0.14, 2.28]	**3.81 [0.99, 15.7]**^c^	0.62 [0.11, 3.90]
Specific IgG4 to dog dander	1.30 [0.60, 2.73]	**1.75 [1.03, 4.63]**^b^	**2.15 [0.98, 4.33]**^c^	1.35 [0.53, 2.48]	0.85 [0.50, 2.18]
Specific IgG4 to cat dander	0.40 [0.10, 0.90]	**0.50 [0.30, 1.03]**^a^	0.30 [0.10, 0.30]	**0.60 [0.20, 1.60]**^b^	0.30 [0.10, 0.80]
Total IgE	142.9 [65.4, 376.6]	191.3 [49.6, 521.7]	**216.8 [86.8, 464.4]**^a^	150.1 [73.1, 373.4]	125.1 [57.4, 267.5]

Data are shown as frequency (%) or median [q1, q3]. Dog and cat allergy definition: subjects who experienced symptoms of allergic rhinitis, allergic conjunctivitis, skin allergy, asthma or cough during contact with dog or cat. Subjects with allergy to dog, cat or both were compared to those without allergy to dog or cat using Fisher's exact test for categorical variables and Wilcoxon's rank-sum test for continuous variables.

a *P* < 0.05

b *P* < 0.01

c *P* < 0.001

### Serum levels of dog- and cat-dander specific IgE, IgG4 and total IgE

One hundred sixty-six individuals out of the 238 tested patients (70%) were proven to be sensitized to both cat and dog by specific IgE measurement (specific IgE ≥0.1 kU_A_/L for both dog and cat dander allergens). Serum concentrations of specific IgE and IgG4 to dog and cat dander in subjects with allergy to both dog and cat were all higher than those in subjects without allergy to dog or cat (dog-specific IgE, 2.49 *vs* 0.26 kU_A_/L, *P* < 0.001; cat-specific IgE, 5.00 *vs* 0.62 kU_A_/L, *P* < 0.001; dog-specific IgG4, 1.75 *vs* 0.85 kU_A_/L, *P* < 0.01; cat-specific IgG4, 0.50 *vs* 0.30 kU_A_/L, *P* < 0.05). In subjects with allergy only to dog, serum concentrations of specific IgE and IgG4 to dog dander but not to cat dancer were higher than those in subjects without allergy to dog or cat (dog-specific IgE, 3.09 *vs* 0.26 kU_A_/L, *P* < 0.001; dog-specific IgG4, 2.15 *vs* 0.85 kU_A_/L, *P* < 0.001). In subjects with allergy only to cat, serum concentrations of specific IgE and IgG4 to cat dander but not to dog dander were higher than those in subjects without allergy to dog or cat (cat-specific IgE, 3.81 *vs* 0.62 kU_A_/L, *P* < 0.001; dog-specific IgG4, 0.60 *vs* 0.30 kU_A_/L, *P* < 0.05). Serum concentration of total IgE was higher in subjects with dog allergy than that in subjects without allergy to dog or cat (216.8 *vs* 125.1 kU_A_/L, *P* < 0.05).

### Sensitization to dog and cat components

**Fig. 2:**
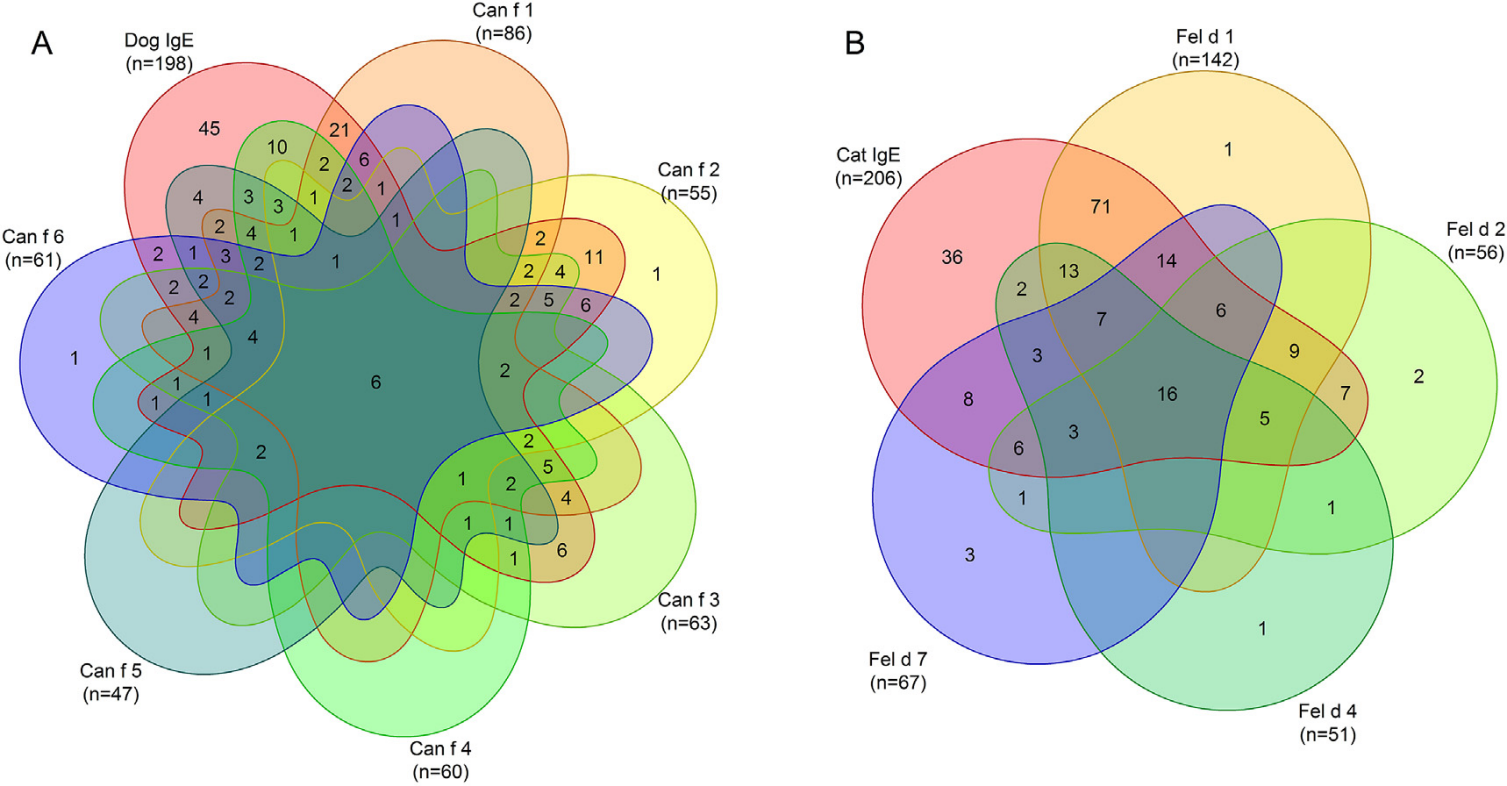
Venn diagram for sIgE antibody test results for pet dander extracts and pet components. If sIgE to pet dander extract were ≥0.1 kU_A_/L, the sIgE antibodies to dog and/or cat components were analyzed. Dog (A) and Cat (B)

Among the dog components, sensitization to Can f 1 was most common (36.1% in whole subjects), followed by sensitization to Can f 3 (26.5%), Can f 6 (25.6%), Can f 4 (22.7%), Can f 2 (22.3%), and Can f 5 (19.7%) (Fig. 2A).

In total, 198 subjects (83.1% in whole subjects) were sensitized to dog dander and 45 of these (23%) were negative on all six dog components. In contrast, 2 subjects were negative for dog dander but positive to a dog component. Among them, one was positive for Can f 2 (0.35 KU_A_/L) and the other for Can f 6 (0.12 kU_A_/L), respectively.

Among the cat components, sensitization to Fel d 1 was most common (59.7% in whole subjects), followed by Fel d 7 (28.2%), Feld d 2 (23.5%) and Fel d 4 (21.4%) (Fig. 2B).

In total, 206 subjects (86.6% in whole subjects) were sensitized to cat dander and 36 of these (17%) were negative on all 4 cat components. In contrast, 9 subjects were negative for cat dander but positive to a cat component. Among them, 4 of them were positive to Fel d 7 and had a higher sIgE value to Can f 1. Three were positive to Fel d 2 and were positive to Can f 3 with a higher value. The remaining 2 were positive for Fel d 4 and Fel d 1, respectively, but with no explanatory cross reactions.

**Fig. 3:**
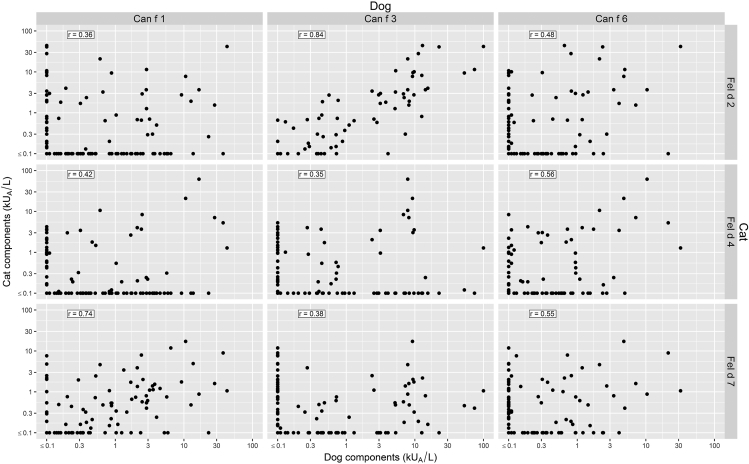
Comparison of binding to Can f 1, Can f 3, Can f 6, Fel d 2, Fel d 4 and Fel d 7 (kUA/L) component allergens within the dog- and cat sensitized population (*n* = 238). *r* = Spearman correlation coefficient. Values < 0.1 were set to 0.1 to be able to plot on log axis, but the correlation coefficients were calculated on unmodified values

Pairwise correlation of IgE levels for cross-reactive allergens was *r* = 0.84 (*P* < 0.001) for Can f 3 and Fel d 2, and *r* = 0.74 (*P* < 0.001) for Can f 1 and Fel d 7 (Fig. 3). The corresponding pairwise comparison of the other combinations between the dog and cat components shown in Fig. 3 correlated significantly (p < 0.05) as well but not at the same level.

### Sensitization in relation to allergy, asthma, and rhinitis symptoms

**Fig. 4:**
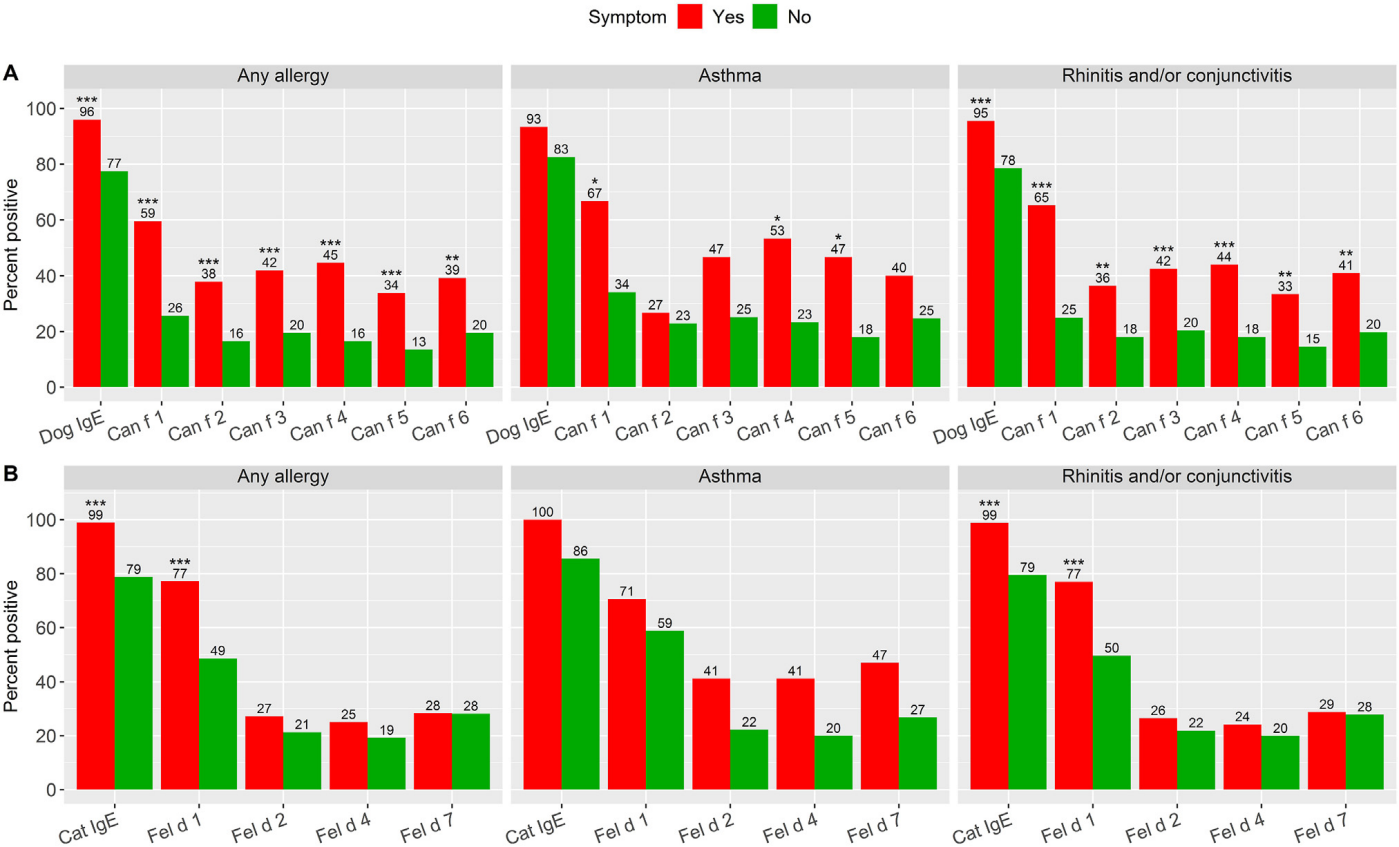
Percentage of subjects positive for each component by symptom (*n* = 238) for dog (A) and cat (B). All subjects were sensitized to both cat and dog respectively. Subset of symptoms of any allergic symptom, asthma symptom and rhinoconjunctivitis symptom. (∗*P* < 0.05, ∗∗*P* < 0.01, ∗∗∗*P* < 0.001. Fisher's exact test of the hypothesis that % Yes = % No)

Sensitization to dog dander extract and to any dog components were significantly associated with allergic symptoms upon contact with dog (Fig. 4). Asthma symptom was specifically associated with sensitization to Can f 1, Can f 4 and Can f 5 (*P* < 0.05) respectively. Asthma symptom was not associated with sensitization to dog dander extract. Rhinoconjunctivitis symptom was associated with sensitization to dog dander extract and to all 6 dog components. Sensitization to cat dander extract and to Fel d 1 were significantly associated with allergic symptoms upon contact with cat. Asthma symptom was not found to be associated with sensitization to cat dander extract or to any of the 4 cat components. Rhinoconjunctivitis symptom was associated with sensitization to cat dander extract and to Fel d 1.

**Fig. 5:**
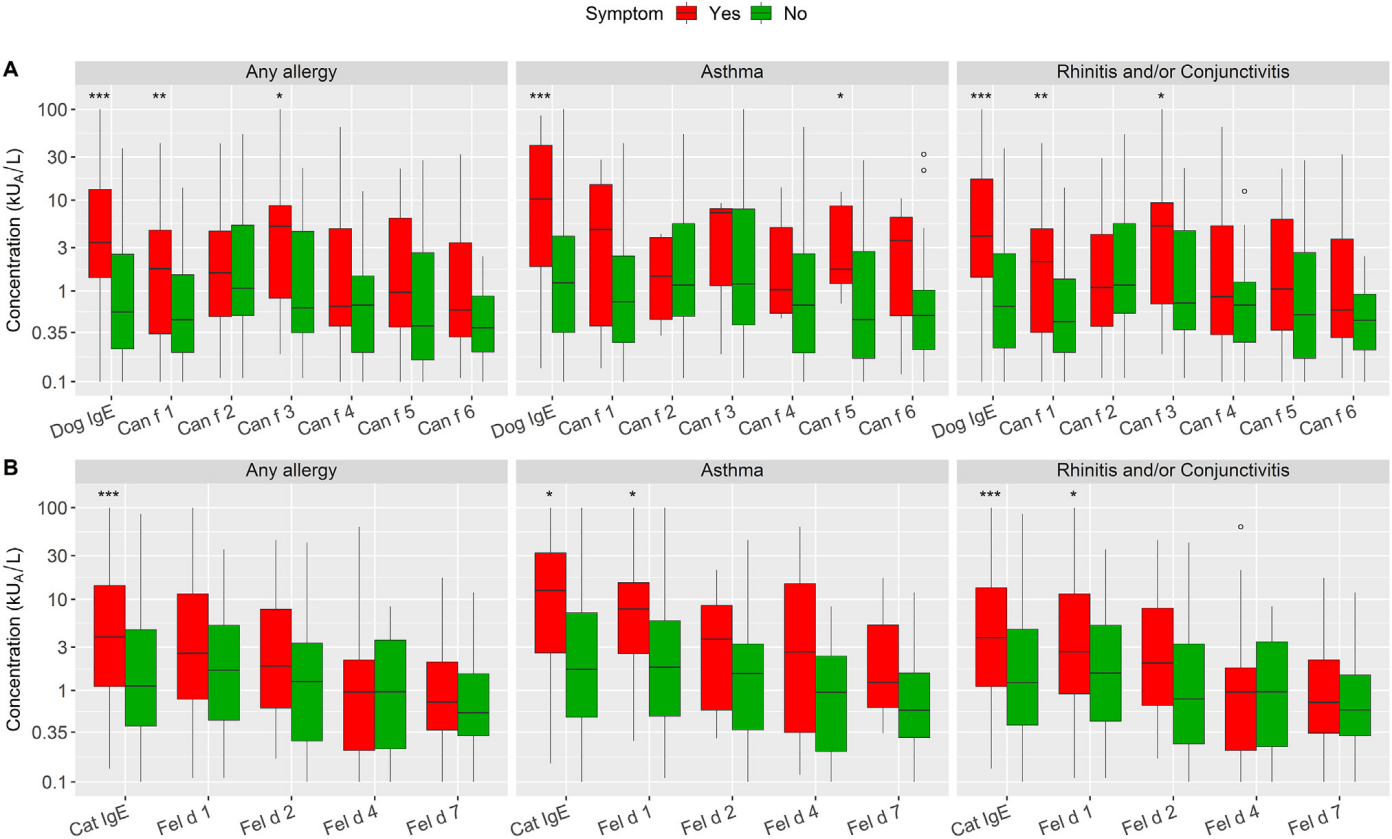
Boxplot of only positive concentrations (≥0.1) by allergic symptom on exposure to Dog (A) and Cat (B). (∗*P* < 0.05, ∗∗*P* < 0.01, ∗∗∗*P* < 0.001. Wilcoxon rank sum (exact) test of the hypothesis that there is no location shift in data distribution between Yes and No). The circle symbol represents an outlier (a value beyond 1.5∗IQR (inter-quartile range) from the 1^st^ and 3^rd^ quartile)

The concentration distribution among all positive tests (≥0.1) split into symptom (yes vs no) is shown in Fig. 5. Overall, there was a large variation in IgE levels against the different components of dog and cat allergen, and the highest levels were found against Can f 3 among dog components and Fel d 1 in cat components. Subjects with any allergic symptoms on cat and dog exposure had significantly higher levels of IgE antibodies to dog- and cat-dander extracts, respectively, (*P* < 0.001) compared to subjects without any symptoms. The same was true for subjects with asthma symptom (*P* < 0.001) and rhinoconjunctivitis symptom (*P* < 0.001) with contact to dog compared to those without symptoms. The same association was found for cat extract (any allergy, asthma, and rhinoconjunctivitis (*P* < 0.001, *P* < 0.05, and *P* < 0.001, respectively). Subjects with asthma symptom upon contact with dog had significantly higher level of IgE to Can f 5 (*P* < 0.05). Subjects with rhinoconjunctivitis symptoms had higher levels of Can f 1 (*P* < 0.01) and Can f 3 (*P* < 0.05). The corresponding association was found for Fel d 1 for asthma symptom and for rhinoconjunctivitis symptom (*P* < 0.05, respectively).

**Fig. 6:**
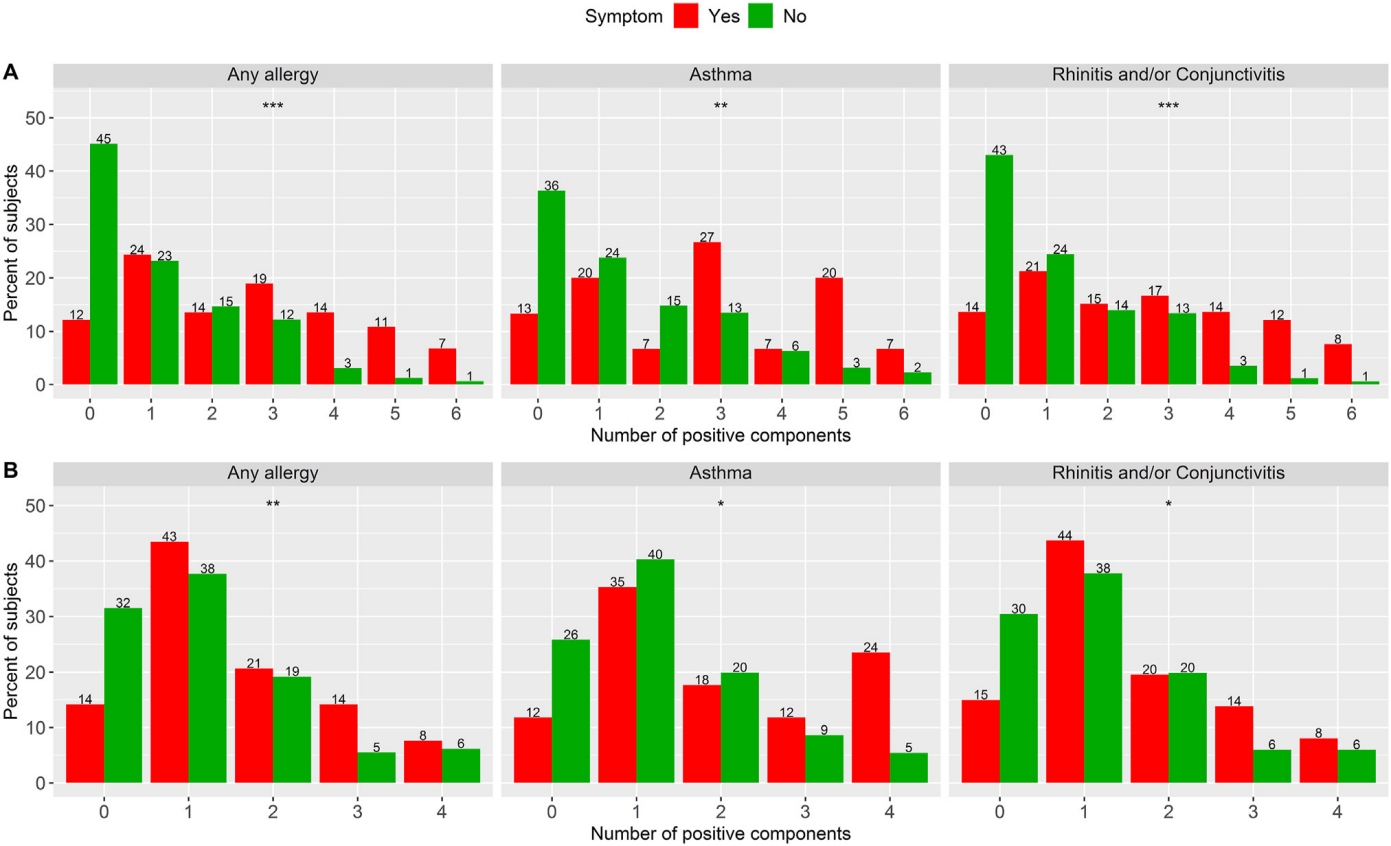
Percentage of subjects with 0–4 positive cat components and 0–6 positive dog components within each allergy group (*n* = 238). Dog (A) and Cat (B). (∗*P* < 0.05, ∗∗*P* < 0.01, ∗∗∗*P* < 0.001. Wilcoxon rank sum (exact) test of the hypothesis that there is no location shift in data distribution between Yes and No. Percent sums to 100 within each symptom outcome (yes and no)

There was a statistically significant association between an increasing number of sensitizing dog components and the likelihood of having any allergic symptoms (*P* < 0.001), asthma symptom (*P* < 0.01) and rhinoconjunctivitis symptom (*P* < 0.001) upon contact with dog; so, was there a statistically significant association between an increasing number of sensitizing cat components and the likelihood of having any allergic symptoms (*P* < 0.01), asthma symptom (*P* < 0.05) and rhinoconjunctivitis symptom (*P* < 0.05) upon contact with cat (Fig. 6).

## Discussion

In this study we investigated the usefulness of molecular allergology as a predictive tool in adults for allergic symptoms on dog and cat exposure. We analyzed IgE to Can f 1 to Can f 6 and Fel d 1, Fel d 2, Fel d 4, and Fel d 7 and explored associations between sensitization pattern and symptoms.

Polysensitization to all 6 dog components and all 4 cat components conferred the overall highest risk of having allergic symptoms, asthma symptom, and rhinoconjunctivitis symptom upon contact with dog and cat. This association between adult multisensitization and allergic symptom on dog and cat exposure confirms and refines results from previous pediatric studies.^18^, ^19^, ^20^, ^21^ Our study also confirms the findings from an adult asthma cohort showing that both specific cat and dog components and their sensitization patterns were associated with substantially increased risk of current asthma, allergic rhinitis, and concomitant asthma and rhinitis.^11^^,^^14^ This increased complexity and serum concentration of IgE antibodies have been called molecular spreading and have been described for grass pollen and mite sensitization.^21^^,^^22^ It is now well documented that asthmatic children are characterized by a more complex molecular pattern of IgE sensitization than non-asthmatic children.^1^^,^^23^^,^^24^ We can now document this molecular spreading among a group of adults with high degree of pet exposure and found a similar association with symptoms. Our population sensitization pattern was like the Swedish asthma population and with a much higher prevalence rate than a random population.^11^^,^^14^ The prevalence sensitization rate to Fel d 1 was 27% compared to 8.9% in the random Swedish population and for Can f 1 16.5% compared with 3.0% respectively. Interesting differences were found for Can f 5 sensitization. It was the most common dog sensitizer in the random Swedish population but was the least common sensitizer in our population. Further, there was no Can f 5 monosensitized subjects in our study in comparison with 33% in the random population and 17% in asthma population of those sensitized to any dog components. Liccardi et al found that 58% of those sensitized to Can f 5 were monosensitized among the allergy patient population in Italy.**^25^** The real significance of Can f 5 in respiratory allergy remains controversial.**^10^** In fact, Käck et al found no association of positive dog nasal provocation test and Can f 5 sensitization in children.**^18^** This is contrast to our findings that sIgE levels to Can f 5 was the only dog component which significantly differed between asthmatics and no asthmatics. This might be an effect of age. However, it is more likely an effect of exposure, since most children in the study were not exposed to dogs at home in contrast to our subjects.

Surprisingly many subjects were sensitized to the dog and cat dander extract but negative on the respective allergen components, 23% and 17%, respectively. This was true even though we had included the new components Can f 4, Can f 6 and Fel d 7 into the test algorithm in order to test for an almost complete component panel. This contrasts with the findings of Tsolakis et al where only 4% of the subjects were sensitized to cat dander but not to any of the analyzed components using the same test algorithm for cat as we did.**^12^** Our finding could be explained by allergen components that was not tested for, such as Fel d 3 (cystatin) and Fel d 8 (latherin-like) for cat.**^26^** According to the European Academy of Allergy and Clinical Immunology (EAACI) Molecular Allergology User's Guide, Can f 1- Can f 6 seem to cover the major and relevant minor allergenic molecules for dog.**^26^** We therefore assume that missing new cat or dog components is a less likely explanation. Another explanation could be the presence of α-Gal in cat and dog dander extracts.**^27^** Kiewiet et al have in a recent study of patients with α-Gal syndrome found a high frequency of sensitization to both dog and cat extract but a low frequency of genuine cat and dog sensitization using CRD.**^28^** Only 21.7% of the cat extract sensitized subjects were sensitized to the major cat allergen, Fel d 1, reflecting genuine cat sensitization. Thus, less than one-third of the cat sensitized patients can be considered as primarily cat sensitized. For dog the difference was even larger, since solely 10.1% of the patients were observed to be sensitized to the dog allergen molecules Can f 1 and Can f 5 mirroring genuine dog sensitization. The conclusion to draw from this study and our study is that sensitization to cat and dog needs to be investigated on a molecular allergen level more extensively, including specific IgE antibodies against galactose-α-1,3-galactose.

We also observed that 9 subjects were negative on cat dander extract but positive for cat components. Four of them were positive to Fel d 7 and 3 were positive to Fel d 2. The explanation for this is most likely lipocalin and albumin cross-reactivity as the subjects were also sensitized to Can f 1 and Can f 3, respectively, with higher IgE concentration. Fel d 7 has high potential to cross-react with Can f 1 with which it shares 62% amino acid identity,**^29^** and we were able to document the homology. Serum albumins are highly abundant and are considered minor allergens with low prevalence of IgE reactivity among patients allergic to its source. Serum albumins remain relevant, because they are responsible for species cross-reactivity due to high sequence identity (up to 82%) and we found in our study high level of cross reactivity between Fel d 2 and Can f 3. It is discussed if patients with IgE to serum albumins should be advised to avoid mammalian pets as they may experience clinical symptoms upon contact with any pet.**^26^** Barber et al have recently published a proposed AIT treatment algorithm for cat and dog allergy which discriminates between primary sensitization and cross-reactive sIgE response.**^30^** Only patients sensitized to major allergens should be eligible for AIT. For example, patients not sensitized to Fel d 1 but to Fel d 2/4/7 do not qualify for cat AIT according to the algorithm.

We found that serum level of dog and cat specific IgG4 respectively was increased in subjects with pet allergy compared with those without it. It has been reported that the measurement of IgG4-ab cannot be used to determine whether a patient is sensitized or not and that IgG4-ab only seem to be part of a physiological response after prolonged antigen exposure.**^31^** We can confirm that IgE sensitization is associated with higher levels of IgG4-ab to cat and dog extract. Meanwhile, we were not able to examine if the IgG4-ab could indicate a protective role against allergic reactions. Our subjects might be highly exposed to dog and cat dander which could explain the high prevalence of both IgE and IgG4 antibodies to these allergens. This indicates that also IgE sensitization reflects exposure. This is in line with the findings by Matsui et al who found significant relationships between cockroach allergen exposure and sensitization.**^32^**

Drawbacks of our study are that we enrolled participants who attended a pet exhibition. This means that study population was selected and not representing a general, random or patient population. We therefore need to interpret our results with caution and not to generalize them. Nearly all subjects in our study were exposed to cats and dogs to a degree and that limits our possibilities to draw conclusions regarding measurements of IgG4. In addition, our study relied on self-reported pet exposure and allergic symptoms. Thus, the possibility of reporting bias cannot be excluded, particularly for study participants whose allergies were not confirmed by doctors. In this survey, we were not able to perform provocation tests in order to confirm the presence of pet allergies.

We conclude that sensitization to specific dog and cat allergen components and their sensitization patterns differ, and that molecular spreading is associated with high likelihood to have allergic symptoms, including those of asthma and rhinoconjunctivitis, upon cat and dog exposure. Identifying those with furry animal allergen component sensitization, particularly those with polysensitization, may help to identify patients at heightened risk of allergic symptom on cat and dog exposure.

## Acknowledgement

We are especially grateful to Hyun Ju Im, Jung Won Kim, Hyun Ae Kim, Jung Mi Ryu, Hyun Jung Park, Ji Yeon Suh, Hye Ryun Song, Young Rim Yoo, Jung Cheol Chang, Myung Ja Lee and Yeo Jin Choe for their devotion to our study.

## Funding

This study was funded by a grant from Korean Academy of Asthma, Allergy, and Clinical Immunology (2018), and from the Ministry of Education through the Basic Science Research Program of the National Research Foundation of Korea (NRF-2015R1D1A1A02061943).

## Availability of data and materials

The authors confirm that the data supporting the findings of this study are available within the article and its supplementary materials.

## Ethics approval

The protocol of this study was reviewed and approved by the Gachon University Gil Medical Center institutional review board (IRB approval number: GAIRB2018-194).

## Author details

Kang S–Y, Yang M − S, Lee S.M., and Lee S.P. involved in study concept and design. Kang S–Y, Yang M − S, and Lee S.M. contributed to the acquisition of data. Borres M.P., Andersson M., and Lee S.M. contributed the analysis and interpretation of data and drafted the manuscript. Kang S–Y, Yang M − S, Borres M.P., Andersson M., Lee S.M., and Lee S.P. performed critical revision of the manuscript for important intellectual content. All authors approved the manuscript for submission.

## Consent for publication

We hereby declare that we all participated in the study and in the development of the manuscript titled “The association between specific IgE antibodies to component allergens and allergic symptoms on dog and cat exposure among Korean pet exhibition participants”. We have read the final version and give our consents for the article to be published in *the World Allergy Organization Journal.*

## Declaration of competing interest

M. P. Borres and M Andersson are employees of Thermo Fisher Scientific (Uppsala, Sweden). SM Lee has received material from Thermo Fisher Scientific to perform the IgE analyses for this work. The rest of the authors declare that they have no relevant conflicts of interest related to this work.
